# Proteinuria and Reduced Estimated Glomerular Filtration Rate are Independently Associated With Lower Cognitive Abilities in Apparently Healthy Community-Dwelling Elderly Men in Japan: A Cross-sectional Study

**DOI:** 10.2188/jea.JE20180258

**Published:** 2020-06-05

**Authors:** Akira Fujiyoshi, Katsuyuki Miura, Takayoshi Ohkubo, Naoko Miyagawa, Yoshino Saito, Itsuko Miyazawa, Akihiko Shiino, Aya Kadota, Sayaka Kadowaki, Takashi Hisamatsu, Sayuki Torii, Naoyuki Takashima, Ikuo Tooyama, Hirotsugu Ueshima

**Affiliations:** 1 Department of Hygiene, Wakayama Medical School, Wakayama, Japan; 2 Department of Public Health, Shiga University of Medical Science, Shiga, Japan; 3 Center for Epidemiologic Research in Asia, Shiga University of Medical Science, Shiga, Japan; 4 Department of Hygiene and Public Health, Teikyo University School of Medicine, Tokyo, Japan; 5 International Center for Nutrition and Information, National Institute of Health and Nutrition, National Institutes of Biomedical Innovation, Health and Nutrition, Tokyo, Japan; 6 Department of Medicine, Shiga University of Medical Science, Shiga, Japan; 7 Molecular Neuroscience Research Center, Shiga University of Medical Science, Shiga, Japan; 8 Department of Environmental Medicine and Public Health, Faculty of Medicine, Shimane University, Shimane, Japan; 9 Department of Public Health, Faculty of Medicine, Kindai University, Osaka, Japan

**Keywords:** cognitive function, proteinuria, estimated glomerular filtration rate, chronic kidney disease (CKD)

## Abstract

**Background:**

The association of proteinuria and reduced estimated glomerular filtration rate (eGFR) with cognition needs more clarification. We cross-sectionally examined whether proteinuria and reduced eGFR, even in moderate stages, were independently associated with lower cognition in a community-based sample of elderly men.

**Methods:**

Our cohort initially comprised 1,094 men aged 40–79 years from a random sample from Shiga, Japan in 2006–2008. Of 853 men who returned for the follow-up examination (2009–2014), we analyzed 561 who were ≥65 years, free of stroke, and completed the Cognitive Abilities Screening Instrument (CASI) at follow-up (higher CASI scores [range 0 to 100] indicate better cognition). Proteinuria was assessed via dipstick. eGFR was calculated according to the Chronic Kidney Disease Epidemiology Collaboration Equation. Participants were divided into three groups either by eGFR (≥60, 59–40, and <40 mL/min/1.73 m^2^) or by proteinuria (no, trace, and positive), considered normal, moderate, and advanced, respectively. Using linear regression, we computed mean CASI score, with simultaneous adjustment for proteinuria and eGFR in addition to other potential confounders.

**Results:**

Significant trends of lower cognition were observed across the groups of worse proteinuria and lower eGFR independently: multivariable-adjusted mean CASI scores were 90.1, 89.3, and 88.4 for proteinuria (*P*_trend_ = 0.029), and 90.0, 88.5, and 88.5 for eGFR (*P*_trend_ = 0.015) in mutual-adjustment model.

**Conclusions:**

Proteinuria and reduced eGFR, even in their moderate stages, were independently associated with lower cognition in a community-based sample of elderly men. The results suggest the importance of proteinuria and low eGFR for early detection and prevention of cognitive decline.

## INTRODUCTION

Early identification of modifiable risk factors for cognitive decline and dementia is an important public health issue because of the global burden of dementia and aging populations worldwide. Chronic kidney disease (CKD), commonly defined based on the presence of albuminuria/proteinuria and/or reduced estimated glomerular filtration rate (eGFR), is considered a potential risk factor for cognitive decline and dementia.^1^ The kidneys and the brain are both susceptible to vascular damage owing to their similarity in anatomic and hemodynamic features, which may explain, in part, the CKD-cognition relationship. However, existing literature remains limited as to whether both proteinuria and eGFR are associated with cognitive impairment. For example, in a recent meta-analysis of population-based studies, albuminuria/proteinuria was most consistently associated with cognitive impairment, but the association of reduced eGFR (defined as <60 mL/min/1.73 m^2^) was weak and inconsistent within the study results.^2^ Other important but unsettled questions include whether moderate stages of albuminuria/proteinuria or reduced eGFR are independently associated with low cognition, and whether individuals with coexisting proteinuria and low eGFR have lower cognition than individuals with either condition alone. These questions have important clinical implications in early detection and prevention of cognitive impairment because the presence of either proteinuria or low eGFR, even in their moderate stages, may alert the clinician and the patient to be more aware of the need to prevent further deterioration of cognitive impairment.

The primary aim of this study was to examine the association of proteinuria and eGFR with cognitive function, separately and jointly, in a community-based sample of elderly men. We hypothesized that the presence of proteinuria and/or lower eGFR are statistically independently associated with lower cognition in a dose-response manner in apparently healthy men. We also investigated whether the coexistence of proteinuria and low eGFR was associated with lower cognition than the presence of either condition alone.

## MATERIALS AND METHODS

### Participants

The Shiga Epidemiological Study of Subclinical Atherosclerosis is a study of subclinical atherosclerosis and its determinants on a sample of Japanese residents. Details of the methods of enrollment have been reported previously.^3^^,^^4^ In brief, from 2006 through 2008, we randomly selected and invited 2,379 Japanese men aged 40–79 years who were residents of Kusatsu City, Shiga, based on the Basic Residents’ Register of the city, to participate in our study. The city, located in central Japan, has an industrial structure similar to the average of that in Japan.^5^ The Register contains information on name, sex, birth date, and address of residents.^6^ A total of 1,094 men agreed to participate in the baseline exam (participation rate, 46%). From 2009 through 2014, all participants were invited to take part in a follow-up exam, and 853 of them agreed.^7^ The study was carried out in accordance with the Code of Ethics of the World Medical Association (Declaration of Helsinki) and approved by the institutional review board of Shiga University of Medical Science. Written informed consent was obtained from all participants. For the present study, using a priori criteria, we excluded those aged <65 years (*n* = 218), with a history of stroke (*n* = 36), and who did not complete the Cognitive Abilities Screening Instrument (CASI) at the follow-up exam (*n* = 32). We further excluded the participants with missing pertinent variables (*n* = 6), leaving 561 men for the final analyses.

### Measurements

Data on medical history, use of medications, smoking, alcohol intake, and other lifestyle factors were collected from each participant using a self-administered questionnaire. Trained technicians confirmed the completed questionnaire with participants. Details on ascertaining smoking habits were reported previously,^5^ and a similar method was used to determine drinking habits. Body weight and height were measured while the participant was wearing light clothing without shoes. Blood pressure was measured using an automated sphygmomanometer (BP-8800; Colin Medical Technology, Komaki, Japan). Two consecutive measurements in the right arm of the seated participant, after sitting quietly for 5 minutes, were taken; the mean of these two measurements was used for analyses. Blood specimens were obtained early in the clinic visit after a 12-hour fast and used for laboratory testing, including lipids and glucose concentrations. Serum creatinine was measured by the enzymatic method (Espa CRE-liquid II; NIPRO, Osaka, Japan). Serum lipid concentrations were determined at a single laboratory (Shiga Laboratory; MEDIC, Shiga, Japan) that had been certified for standardized lipid measurements according to the protocols of the United States Centers for Disease Control and Prevention/Cholesterol Reference Method Laboratory Network.^8^ Low-density lipoprotein (LDL) cholesterol concentrations were calculated using the Friedewald equation (In the case of triglycerides concentration >400 mg/dL, we treated them as missing).^9^ Glycated hemoglobin (HbA1c) was measured using latex agglutination inhibition assay according to either the Japan Diabetes Society (JDS) protocol or that of the National Glycohemoglobin Standardization Program (NGSP).^10^ JDS values were converted to NGSP values using the equation recommended by JDS^10^: NGSP value (%) = 1.02 × JDS value (%) + 0.25. We administered the 6-item Kessler Psychological Distress Scale (K6),^11^ a commonly used tool for the assessment of mood. The K6 scale ranges from 0 to 24, with higher scores indicating the presence of a mood disorder, including depression. The validity of the K6 for a Japanese population is reported elsewhere.^12^

### Cognitive function

Cognitive function was evaluated by participants’ performance on CASI (Version J-1.0), a validated comprehensive measure of intellectual function comprised of 25 questions.^13^ Performance scores (CASI score) range from 0 to 100, with higher scores indicating better cognitive function. CASI scores <74 correspond to a score of <22 on Folstein’s Mini-Mental State Examination, raising the possibility of dementia.^14^ Three raters (AF, NM, and YS) independently determined the CASI score based on recorded responses from participants. The intraclass correlation coefficient across the raters was 0.977 based on recorded samples of 20 participants.

### Assessment of proteinuria and eGFR

Proteinuria was assessed using dipstick (Uriace; Terumo, Tokyo, Japan). For eGFR, we used the CKD Epidemiology Collaboration (CKD-EPI) equation modified for the Japanese in the main analysis. The equation is as below^15^^,^^16^:

$$\begin{array}{l} \text{eGFR}=141\times (\text{s-Cr/0.9}){}^{(-0.411)}\times 0.993^{\text{(Age)}}\times 0.813,\\ \text{ for those with s-Cr ≤}0.9\;\text{mg/dL}\;(79.6\;µ\text{mol/L}) \end{array}$$

$$\begin{array}{l} \text{eGFR}=141\times (\text{s-Cr/0.9}){}^{(-1.209)}\times 0.993^{\text{(Age)}}\times 0.813,\\ \text{ for those with s-Cr >}0.9\;\text{mg/dL }(79.6\;µ\text{mol/L}), \end{array}$$

where s-Cr stands for serum creatinine. We chose the CKD-EPI equation over the Modification of Diet in Renal Disease (MDRD)-based equations because of its superior accuracy,^17^^,^^18^ with less bias at GFR ≥60 mL/min/1.73 m^2^,^19^ and better predictability of cardiovascular and other outcomes^20^ shown in multiple populations, including Japanese ones,^15^^,^^21^ compared to the latter. In a sensitivity analysis, we replaced the CKD-EPI Equations with the MDRD-based equation,^22^ which is used in the 2018 clinical practice guidelines by the Japanese Society of Nephrology (JSN),^23^ as formulated below:

$$\begin{array}{l} {\text{eGFR}}_{\text{creat}}\;[\text{mL/min/1.73}\;{\text{m}}^{\text{2}}]\\ \;=\text{194}\times \text{s-Cr }[\text{mg/dL}]{}^{(-1.094)}\times \text{Age }[\text{year}]{}^{(-0.287)}. \end{array}$$

We defined low eGFR as eGFR <60 mL/min/1.73 m^2^, proteinuria as trace or greater degree of proteinuria, and CKD as either having low eGFR or proteinuria.

### Statistical analysis

In the main analysis, we divided participants into categories according to eGFR levels of ≥60, 59 to 40, and <40 [mL/min/1.73 m^2^], and proteinuria as absent (−), trace (±), and positive (≥1+), operationally considering each category as normal, moderate, and advanced stage, respectively. The eGFR cutoff of <40 mL/min/1.73 m^2^ was chosen somewhat arbitrarily. At the inception of the study we planned to use cutoff of <30 mL/min/1.73 m^2^ as it defines advanced reduction of eGFR in some guidelines^23^^,^^24^ and we had a concern that use of a higher eGFR cutoff, such as 45, may introduce more variation in cognition level relative to the group of people with eGFR <30. However, owing to the fact that few participants (*n* = 2) had an eGFR <30 mL/min/1.73 m^2^, we have elected to use 40 mL/min/1.73 m^2^ for the cutoff to balance between maintaining a reasonable sample size versus observing enough effect of reduced eGFR on cognition. Hypertension was defined as systolic/diastolic blood pressure ≥140/90 mm Hg or medication use. Diabetes mellitus was defined as fasting blood glucose ≥7.0 mmol/L (≥126 mg/dL) or HbA1c (NGSP) ≥6.5% or medication use. Dyslipidemia was defined as LDL cholesterol concentration ≥3.6 mmol/L (140 mg/dL),^25^ high-density lipoprotein (HDL) cholesterol concentration <1.0 mmol/L (40 mg/dL), or dyslipidemia medication use.

Linear regression was used to obtain adjusted mean CASI score treating explanatory categories (eGFR and proteinuria) as nominal. For confirmation, we first examined whether the presence of CKD was associated with lower CASI score in reference to the absence of CKD after multivariable adjustment. Second, we examined potential dose-response by inserting categories of either proteinuria (no, trace, positive: nominal) or eGFR (<40, 40–59, ≥60 mL/min/1.73 m^2^: nominal), separately as an explanatory variable (single adjustment model). Third, we simultaneously included the categories of proteinuria and eGFR to examine the mutual independence in relationship to cognition (mutual adjustment model). Finally, for the secondary aim, we categorized participants into three groups based on the status of low eGFR (<60 mL/min/1.73 m^2^) and proteinuria (trace or more): “No CKD” represented having neither low eGFR nor proteinuria, “isolated low eGFR or proteinuria” represented having either low eGFR or proteinuria but not both, and “low eGFR and proteinuria” represented having both conditions. Then we compared adjusted CASI scores among the three groups. *P*-values for trends were computed treating the categories as ordinal. In post-hoc analysis, we further repeated the analysis according to four nominal categories by separating isolated low eGFR and isolated proteinuria considering that they may have different pathophysiology from each other. We adjusted for the following covariates throughout the models: age (years), highest education year attained (years),^26^^–^^28^ drinking and smoking habits (current/past/never), body mass index (kg/m^2^), hypertension (yes/no), diabetes mellitus (yes/no), dyslipidemia (yes/no), and hemoglobin concentration (g/dL). In a post-hoc sensitivity analysis, we explored whether adding the K6 score to the model affected the main results. In a separate post-hoc sensitivity analysis, we replaced eGFR cutoff of 40 with 45 mL/min/1.73 m^2^ and repeated an analysis parallel to the main one. Values of *P* < 0.05 were considered significant, and all analyses were two-tailed. SAS version 9.4 software (SAS Institute, Cary, NC, USA) was used.

## RESULTS

Of 561 participants, 270 men (48.1%) met the definition of CKD either by isolated proteinuria or isolated low eGFR (*n* = 224) or by the presence of both (*n* = 46). Table 1 shows characteristics of participants according to proteinuria status (eTable 1 shows the characteristics according to eGFR category). The mean values for age, eGFR, and CASI score were 72.0 (standard deviation [SD], 4.4) years, 67.5 (SD, 19.1) mL/min/1.73 m^2^, and 89.8 (SD, 5.9), respectively. The number of participants who had no, trace, or positive (≥1+) proteinuria was 343 (61.1%), 189 (33.7%), and 29 (5.2%), respectively. The number of participants who had eGFR of ≥60, 59–40, and <40 mL/min/1.73 m^2^ were 463 (82.5%), 84 (15.0%), and 14 (2.5%), respectively (eTable 1). Two participants had eGFR <30 mL/min/1.73 m^2^ either by the CKD-EPI equation or the JSN equation, and only one reported receiving regular dialysis. Those who were in a higher category of proteinuria tended to be younger, have diabetes, and have lower eGFR and lower CASI scores. Participants with CKD had a mean CASI score of 89.1, which was 1.26 points lower than those without CKD after multivariable adjustment (*P* < 0.01, eTable 2). The magnitude of the difference was equivalent to more than a 3-year difference in age (ie, CASI score of 0.38/age × 3 ≈ 1.26) in the model.

**Table 1.** Demographics of men (≥65 years and free of stroke) according to degree of proteinuria, examined in 2009–2014, Shiga, Japan

	Total (*N* = 561)		Urinary Protein (dipstick)			*P*^a^

			[−]	[+/−]	[≥1+]	
			(*n* = 343)	(*n* = 189)	(*n* = 29)	
Age, years	72.0	(4.4)	72.2	71.8	70.2	0.03
Education, years	12.5	(2.4)	12.5	12.6	12.1	0.79
Body mass index, kg/m^2^	23.1	(2.9)	23.0	23.2	23.3	0.39
Systolic blood pressure, mm Hg	133	(16.5)	133	132	136	0.66
LDL-cholesterol, micromol/L	3.03	(0.76)	3.05	3.02	2.89	0.36
mg/dL^b^	117	(29.2)	118	117	112	
HDL-cholesterol, micromol/L	1.53	(0.43)	1.54	1.53	1.44	0.34
mg/dL^b^	59	(16.6)	60	59	56	
HbA1c, NGSP, %	6.0	(0.9)	5.9	6.1	5.8	0.30
Smoking, %						
current	16.6		15.2	17.5	27.6	0.26
past	63.8		63.8	67.2	41.4	
never	19.6		21.0	15.3	31.0	
Drinking, %						
current	78.1		79.6	76.2	72.4	0.24^c^
past	5.7		5.8	5.3	6.9	
never	16.2		14.6	18.5	20.7	
Hypertension, %	62.4		60.6	63.0	79.3	0.11
Dyslipidemia, %	47.8		46.4	50.8	44.8	0.57
Diabetes mellitus, %	25.8		22.2	31.7	31.0	0.02
Hemoglobin, g/dL	14.4	(1.6)	14.4	14.3	14.4	0.39
K6 scale^d^	8.7	(3.0)	8.6	9.1	8.2	0.31
Serum creatinine, micromol/L	79.6	(18.6)	77.8	80.4	94.6	<0.01
mg/dL^e^	0.9	(0.2)	0.88	0.91	1.07	
eGFR, mL/min/1.73 m^2^	67.5	(19.1)	68.1	67.0	63.0	0.01
CASI score	89.8	(5.9)	90.1	89.3	88.2	0.05

BMI, body mass index; eGFR, estimated glomerular filtration rate; HDL, high-density lipoprotein; K6, 6-item Kessler Psychological Distress Scale; LDL, low-density lipoprotein; NGSP, National Glycohemoglobin Standardization Program; CASI, the Cognitive Abilities Screening Instrument.

CASI score ranges from 0 to 100, with scores <74 raising the possibility of dementia; eGFR estimates were computed using serum creatinine concentration according to the CKD Epidemiology Collaboration (CKD-EPI) Equation modified for the Japanese; Hypertension: defined as systolic/diastolic blood pressure ≥140/90 mm Hg or medication use; Diabetes mellitus: defined as fasting glucose ≥7.0 mmol/L (126 mg/dL) or HbA1c [NGSP] ≥6.5% or medication use; Dyslipidemia: defined as LDL-cholesterol ≥3.6 mmol/L (140 mg/dL) or HDL-cholesterol <1.0 mmol/L (40 mg/dL) or medication use.

^a^*P*-values were computed using linear regression treating proteinuria category as ordinal for continuous variable, and by Mantel Haenzel test for linear trend.

^b^Conversion unit for serum low-density cholesterol and high-density cholesterol from mmol/L to mg/dL is 38.61.

^c^Categories of drinking were collapsed to current vs non-current because of too few past drinkers.

^d^Four participants had missing scales.

^e^Conversion unit for serum creatinine from micromol/L to mg/dL is 0.0113.

### Main analyses

**Figure 1.**
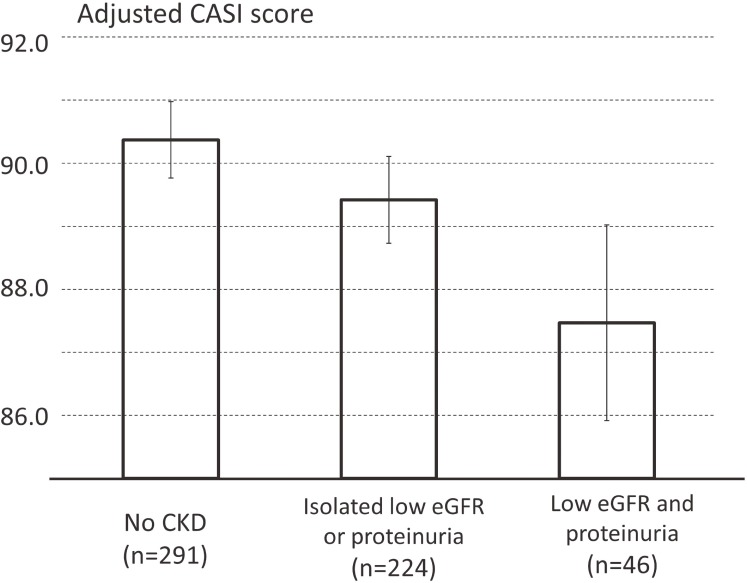
Multivariable-adjusted mean CASI score according to the presence or absence of low eGFR and proteinuria in men aged ≥65 years who were free of stroke (*N* = 561, 2009–2014, Shiga, Japan). Each bar and whiskers represent multivariable-adjusted mean CASI score and 95% confidence intervals. The set of adjusting covariates was same as in the main analyses: age (years), highest education attained (years), drinking/smoking habit (current/past/never), body mass index (kg/m^2^), hypertension (yes/no), diabetes mellitus (yes/no), dyslipidemia (yes/no), and hemoglobin (g/dL). Adjusted mean CASI scores in the three groups were statistically significantly different from each other: *P*-values for pairwise comparison were 0.04 between “no CKD” and “isolated eGFR or proteinuria”, <0.01 between “no CKD” and “low eGFR and proteinuria”, and 0.03 between “isolated eGFR or proteinuria” and “low eGFR and proteinuria”.

Table 2 shows crude and adjusted mean CASI score according to categories of proteinuria and eGFR. In single adjustment models, a higher degree of proteinuria or lower eGFR had a significant trend with lower CASI score in a dose-response fashion, after controlling for potential confounders (*P* for trends were 0.011 and 0.006, respectively). The trends remained statistically significant for both proteinuria and eGFR even after the mutual adjustment (*P* for trends were 0.029 and 0.015, respectively) with slight attenuations across each category of proteinuria or eGFR. Table 3 shows crude CASI score and characteristics according to three groups: “no CKD” (*n* = 291, 51.9%), “isolated low eGFR or proteinuria” (224, 39.9%), and “low eGFR and proteinuria” (46, 8.2%). Participants with “low eGFR and proteinuria” tended to be older, obese, and have hypertension, diabetes, lower concentrations of HDL cholesterol and hemoglobin, and lower CASI scores. Figure 1 shows that multivariable-adjusted mean CASI score was high in the order of “No CKD” (adjusted mean CASI score, 90.4; 95% confidence interval [CI], 89.8–91.0), “isolated low eGFR or proteinuria” (89.4; 95% CI, 88.7–90.1), and “low eGFR and proteinuria” (87.5; 95% CI, 86.0–89.1). The scores of the three groups were significantly different from each other (see Figure 1 legend). In post-hoc analysis separating isolated proteinuria (*n* = 172) and isolated low eGFR (*n* = 52), crude mean CASI score for each of two category was 89.9 and 88.7, respectively. Multivariable-adjusted mean CASI score was high, by point estimate, in the order of “no CKD”, “isolated proteinuria”, “isolated low eGFR”, and “low eGFR and proteinuria.” However, there was no statistical difference in the adjusted mean CASI score between isolated proteinuria and isolated eGFR (eTable 3).

**Table 2.** Crude and multivariable-adjusted mean CASI score according to proteinuria and/or eGFR category in men aged ≥65 years who were free of stroke (*N* = 561, 2009–2014, Shiga, Japan)

		*n*	crude score mean (SD)	Single adjustment^a^		Mutual adjustment^b^	
	
				score (95% CI)	trend *P*	score (95% CI)	trend *P*
Proteinuria	no	343	90.1 (5.4)	90.2 (89.6, 90.7)		90.1 (89.6, 90.7)	
	trace	189	89.3 (6.7)	89.2 (88.5, 90.0)	0.011	89.3 (88.5, 90.0)	0.029
	≥(1+)	29	88.2 (6.7)	88.1 (86.2, 90.1)^*^		88.4 (86.4, 90.4)	

eGFR^c^, mL/min/1.73 m^2^	≥60	463	90.2 (5.5)	90.1 (89.6, 90.5)		90.0 (89.5, 90.5)	
	59–40	84	88.0 (7.1)	88.4 (87.2, 89.5)^*^	0.006	88.5 (87.3, 89.6)^*^	0.015
	<40	14	85.6 (8.0)	88.0 (85.1, 90.8)		88.5 (85.6, 91.4)	

CASI, Cognitive Abilities Screening Instrument; CI, confidence interval; eGFR, estimated glomerular filtration rate; SD, standard deviation.

CASI is scored 0–100, with scores <74 raising the possibility of dementia; Hypertension: defined as systolic/diastolic blood pressure ≥140/90 mm Hg or medication use; Diabetes mellitus: defined as fasting glucose ≥7.0 mmol/L (126 mg/dL) or HbA1c [NGSP] ≥6.5% or medication use; Dyslipidemia: defined as LDL-cholesterol ≥3.6 mmol/L (140 mg/dL) or HDL-cholesterol <1.0 mmol/L (40 mg/dL) or medication use. In all adjustment, the following covariates were included: age (years), highest education attained (years), drinking/smoking habit (current/past/never), body mass index (kg/m^2^), hypertension (yes/no), diabetes mellitus (yes/no), dyslipidemia (yes/no) and hemoglobin (g/dL).

^a^In single adjustment model, either proteinuria (no/trace/≥1+) or eGFR-category (<40/40–59/≥60 mL/min/1.73 m^2^) was included.

^b^In mutual adjustment model, both proteinuria and eGFR were included.

^c^Estimates were computed using serum creatinine concentration according to the CKD Epidemiology Collaboration (CKD-EPI) Equation modified for the Japanese.

^*^Statistically significant (*P* < 0.05) as compared to normal category (either no proteinuria or eGFR ≥60 mL/min/1.73 m^2^).

**Table 3.** Crude CASI score and characteristics according to presence or absence of low eGFR and proteinuria in men aged ≥65 years who were free of stroke (*N* = 561, 2009–2014, Shiga, Japan)

	No CKD	Isolated low eGFR or proteinuria	Low eGFR and proteinuria	*P*^a^

*N*	291	224	46	
Age, years	72.0	71.7	73.7	0.02
Education, years	12.4	12.6	12.1	0.42
Body mass index, kg/m^2^	23.0	23.1	24.3	0.01
Systolic BP, mm Hg	133	132	135	0.57
LDL-cholesterol, micromol/L	3.05	3.04	2.87	0.30
mg/dL^b^	118	118	111	
HDL-cholesterol, micromol/L	1.56	1.52	1.40	0.03
mg/dL^b^	60	59	54	
HbA1c, NGSP, %	5.9	6.0	6.1	0.26
Smoking, %				
current	15.5	19.2	10.9	0.64
past	64.6	61.6	69.6	
never	19.9	19.2	19.6	
Drinking, %				
current	79.7	77.2	71.7	0.72
past	5.8	5.4	6.5	
never	14.4	17.4	21.7	
Hypertension, %	59.8	60.7	87.0	<0.01
Dyslipidemia, %	45.0	49.6	56.5	0.11
Diabetes mellitus, %	22.3	27.7	39.1	0.04
Hemoglobin, g/dL	14.5	14.3	13.9	0.04
K6 scale^c^	8.6	8.7	9.6	0.10
Serum creatinine, micromol/L	73.4	80.4	112.3	<0.01
mg/dL^d^	0.83	0.91	1.27	
eGFR, mL/min/1.73 m^2^	71.1	66.8	48.1	<0.01
CASI score	90.4	89.6	86.5	<0.01

BMI, body mass index; CASI, Cognitive Abilities Screening Instrument; eGFR, estimated glomerular filtration rate; HDL, high-density lipoprotein; K6, 6-item Kessler Psychological Distress Scale; LDL, low-density lipoprotein; NGSP, National Glycohemoglobin Standardization Program.

CASI scores ranges from 0 to 100, with scores <74 raising the possibility of dementia; Estimates were computed using serum creatinine concentration according to the CKD Epidemiology Collaboration (CKD-EPI) Equation modified for the Japanese; Hypertension: defined as systolic/diastolic blood pressure ≥140/90 mm Hg or medication use; Diabetes mellitus: defined as fasting glucose ≥7.0 mmol/L (126 mg/dL) or HbA1c [NGSP] ≥6.5% or medication use; Dyslipidemia: defined as LDL-cholesterol ≥3.6 mmol/L (140 mg/dL) or HDL-cholesterol <1.0 mmol/L (40 mg/dL) or medication use. Values are crude mean unless otherwise specified. Low eGFR was defined as eGFR <60 mL/min/1.73 m^2^. Proteinuria was defined as trace or higher degree of proteinuria.

^a^*P*-values were computed using linear regression treating three categories (“No CKD”, “Isolated low eGFR or proteinuria”, and “Low eGFR and proteinuria”) as ordinal.

^b^Conversion unit for serum low-density cholesterol and high-density cholesterol from mmol/L to mg/dL is 38.61.

^c^Four participants had missing scales.

^d^Conversion unit for serum creatinine from micromol/L to mg/dL is 0.0113.

### Sensitivity analyses

Replacing the CKD-EPI equation-based eGFR with that by the JSN equation resulted in fewer participants categorized as eGFR ≥60 mL/min/1.73 m^2^ (*n* = 463 by the CKD-EPI equation versus 404 by the JSN equation). Otherwise, however, the results were similar to the main ones (eTable 4). Excluding those participants with eGFR ≤30 mL/min/1.73 m^2^ (*n* = 2) or use of model including K6 scale did not change the findings of the main analyses materially (data not shown). Use of eGFR cutoff of <45 instead of <40 mL/min/1.73 m^2^ resulted in attenuation of graded relationship between CASI score and eGFR category to no significance (*P* trend = 0.069 in mutual adjustment model, eTable 5). The alternatively defined moderate eGFR category (45–59 mL/min/1.73 m^2^), however, remained to be associated with significantly lower adjusted CASI score compared to normal eGFR category.

## DISCUSSION

In this population-based, cross-sectional study of apparently healthy, elderly Japanese men, we found that proteinuria and reduced eGFR were independently associated with lower cognitive function in a graded manner in single adjustment models. The association was independent of conventional vascular risk factors and hemoglobin concentrations. To our knowledge, the present study is the first community-based study that showed dose-response relationships with both proteinuria and low eGFR in relation to cognitive function, although the graded relation with low eGFR was somewhat sensitive to cutoff value. Furthermore, the overall trend remained similar and statistically significant after mutual adjustment for proteinuria and low eGFR.

Identifying risk factors for cognitive decline/dementia is of utmost importance given the rapidly aging populations globally. However, the relationship of proteinuria and/or reduced eGFR with cognitive function needs more clarification. A meta-analysis published in 2012 showed elevated odds of cognitive decline in individuals with CKD than those without CKD,^1^ but only one^29^ of 10 studies assessed in the meta-analysis included proteinuria (albuminuria) in their definitions of CKD. A more recent meta-analysis evaluated the associations of cognitive impairment with proteinuria/albuminuria and eGFR separately. It concluded that proteinuria/albuminuria was most consistently associated with cognitive impairment, consistent with other meta-analysis,^30^ but the association of reduced GFR (defined as <60 mL/min/1.73 m^2^) was weak and inconsistent.^2^ Furthermore, many previous studies used only dichotomized category of CKD (presence/absence), and a dose-response relationship of eGFR or proteinuria with cognition was not consistently documented.^31^ For example, the Hisayama Study, one of the best-known prospective cohort studies in Japan, recently reported a dose-response relationship between baseline albuminuria and risk of dementia (all-cause, Alzheimer, and vascular type).^32^ However, they observed a marginal association of reduced eGFR, defined as <60 mL/min/1.73 m^2^ using a similar method to ours, only with vascular dementia but not with Alzheimer dementia, and they did not assess dose-response relation of eGFR.^32^ Other longitudinal studies, such as the Northern Manhattan Study,^33^ showed a significant relation of reduced eGFR and cognitive decline, whereas others reported no clear relationship between them.^34^ In our study, we observed a significant inverse graded trend of proteinuria, and a significantly lower CASI score in participants with an eGFR of 40–59, or 45–59, mL/min/1.73 m^2^ as compared to those with normal eGFR (≥60 mL/min/1.73 m^2^). These findings have an important implication, as they suggest that the presence of either moderate stage of proteinuria or that of low eGFR should alert a clinician and a patient of the potential presence of impaired cognition that may require intervention to prevent further deterioration. Another important finding of the study is that participants with coexisting proteinuria and low eGFR had the lowest CASI score followed by those with isolated proteinuria or low eGFR compared to participants without CKD. This finding is expected because such coexistence often indicates more advanced renal dysfunction. However, only a few epidemiologic studies, to our knowledge, have evaluated a joint association of proteinuria and reduced eGFR with cognition/dementia.^32^ The finding suggests that the coexistence of proteinuria and reduced eGFR indicates elevated risk of dementia even in apparently healthy men with no history of stroke.

The mechanism linking proteinuria or reduced GFR and cognitive impairment is not well understood. Proteinuria and low eGFR are robust predictors for vascular disease and all-cause death.^35^ Many studies reported a close relationship between CKD and subclinical brain pathologies, including small vessel diseases.^31^^,^^36^ This is not surprising given the common anatomic and vaso-regulatory features of the brain and the kidneys, low-resistant end organs that are susceptible to vascular damage, as both organs receive high-volume blood flow.^31^ Individuals with proteinuria or reduced GFR are likely to have subclinical small vessel disease in the brain,^36^^–^^38^ which is believed to play a causal role in cognitive impairment and dementia.^39^ Another potential explanation would be shared risk factors between CKD and impaired cognition such as hypertension, diabetes mellitus, and smoking.^40^^,^^41^

There are other potential mechanisms that may explain the association at least in part. For example, anemia and depression are common in patients with CKD, and may lower cognition. However, we adjusted for hemoglobin concentrations in our main models, and adding the K6 scale to the models did not materially alter our results. Other conditions that may be common in CKD patients and associated with low cognition include polypharmacy (with or without hypnotics) and subclinical/undiagnosed hypothyroidism.^42^ However, we did not assess those conditions, so we cannot rule out potential confounding by them.

Our sample was randomly selected from apparently healthy community-based elderly men from a city in Japan, and approximately one third and one quarter of the studied participants had hypertension and diabetes, respectively. Probable etiologies of proteinuria or low eGFR in our sample, therefore, include hypertension and diabetes, the main causes of CKD in high- to middle-income countries worldwide,^43^ rather than less common pathologies, such as chronic glomerular nephritis. Hence, our results may not be applicable to a group of patients with uncommon etiologies of proteinuria or reduced eGFR, although we do not have formal clinical diagnoses in our participants categorized to CKD.

We used a dipstick for assessment of proteinuria, which is less accurate than other measures, such as urine albumin-to-creatinine ratio (ACR) or quantified measure of proteinuria via 24-hour urine collection. However, at a population level, dipstick-assessed proteinuria was shown to have a graded relationship with cardiovascular and all-cause mortalities comparable to ACR,^35^ suggesting that dipstick assessment is reasonably reliable for the assessment of proteinuria in epidemiological studies.

The observed difference in CASI score appears to be small. However, given our population-based sample being relatively healthy, in which overall cognition level was preserved and only two participants had eGFR ≤30 mL/min/1.73 m^2^, it should not minimize the impact of proteinuria or low eGFR for early identification of cognitive impairment. The observed graded relationship with eGFR was attenuated in the sensitivity analysis that replaced the eGFR cutoff of 40 with 45 mL/min/1.73 m^2^. This attenuation is not surprising because use of higher eGFR cutoff in categorizing the lowest eGFR group may introduce more variation in cognitive level, especially when considering a small number of participants in our sample who had a low eGFR. The replacement with cutoff of 45, however, did not change our finding that the moderately low eGFR (defined as either 59 to 40, or 59 to 45 mL/min/1.73 m^2^) group had a significantly lower adjusted mean CASI score compared to normal eGFR group. Moreover, the replacement had no effect on the observed independent relationship between proteinuria and cognition (Table 2 and eTable 5). Further studies with more participants having reduced eGFR are needed to obtain more stable estimates.

Certain limitations should be considered when interpreting our findings. First, the results may not be applicable to women because we studied only men. Second, we evaluated proteinuria and eGFR only once on the assumption that the observed results persisted, which perhaps led to more misclassifications in our categorizations of proteinuria and eGFR. However, it may bias our inference toward the null, underestimating the true association, if such misclassifications were random. Third, our outcome was a one-time measure of cognitive function, not a decline of it, which is a criterion of the common definition of dementia. However, studies show that cognitive function assessed at one point time predicts future dementia.^44^^–^^46^ Finally, given the observational and cross-sectional nature of the study, we were unable to prove the direction of temporality of the observed association between exposure (kidney function) and cognition. Strengths of our study include the population-based enrollment, use of a standardized protocol in evaluating outcomes and exposures, and adjustment for covariates.

### Conclusions

We found that proteinuria and reduced eGFR were independently associated with lower cognitive function in a graded fashion, and that the coexistence of these conditions was associated with the lowest cognitive function compared to other groups in a community-based sample of elderly men. The results underscore the importance of proteinuria and reduced eGFR for early detection and prevention of cognitive decline in a general population of elderly men.

## APPENDIX A. SUPPLEMENTARY DATA

eTable 1. Demographics of men (≥65 years and free of stroke) according to eGFR category, examined in 2009–2014, Shiga, Japan

eTable 2. Multivariable-adjusted mean slope of CASI score in men aged ≥65 years who were free of stroke (*N* = 561, 2009–2014, Shiga, Japan)

eTable 3. Crude and adjusted mean CASI score according to four categories by proteinuria and eGFR in men aged ≥65 years who were free of stroke (*N* = 561, 2009–2014, Shiga, Japan)

eTable 4. Crude and multivariable-adjusted mean CASI score according to proteinuria and/or eGFR (by 2012 Japanese Society of Nephrology equation) category in men aged ≥65 years who were free of stroke (*N* = 561, 2009–2014, Shiga, Japan)

eTable 5. Crude and multivariable-adjusted mean CASI score according to proteinuria and/or eGFR category (using cutoff of 45) in men aged ≥65 years who were free of stroke (*N* = 561, 2009–2014, Shiga, Japan)

eAppendix 1. Members of the Shiga Epidemiological Study of Subclinical Atherosclerosis (SESSA) Research Group
