# Supplementary material for: Proteinuria and Reduced Estimated Glomerular Filtration Rate are Independently Associated With Lower Cognitive Abilities in Apparently Healthy Community-Dwelling Elderly Men in Japan: A Cross-sectional Study
Source: J Epidemiol. 2020 Jun 5;30(6):244–52. doi: 10.2188/jea.JE20180258 (PMC7217691; doi:10.2188/jea.JE20180258)
Supplement: Supplementary file 1 [file je-30-244-s001.pdf]

Online Supplementary Material

eTable 1. Demographics of men (≥65 years and free of stroke) according to eGFR category, examined in 2009–2014, Shiga, Japan

	Total (N=561)		eGFR category ($mL/min/1.73\ m^2$)			P^a
			[≥60] (n=463)	[59–40] (n=84)	[<40] (n=14)	
Age, years	72.0	(4.4)	71.6	73.6	74.4	<0.01
Education, years	12.5	(2.4)	12.5	12.7	10.9	0.28
Body mass index, kg/m^2	23.1	(2.9)	23.0	23.7	24.8	<0.01
Systolic blood pressure, mmHg	133	(16.5)	133	133	138	0.48
LDL-cholesterol, micromol/L	3.03	(0.76)	3.05	3.02	2.66	0.17
mg/dL[b]	117	(29.2)	118	117	103	
HDL-cholesterol, micromol/L	1.53	(0.43)	1.56	1.45	1.22	<0.01
mg/dL[b]	59	(16.6)	60	56	47	
HbA1c, NGSP, %	5.97	(0.85)	5.97	6.00	6.03	0.70
Smoking, %						
current	16.6		17.5	10.7	21.4	0.36[c]
past	63.8		63.7	66.7	50.0	
never	19.6		18.8	22.6	28.6	
Drinking, %						
current	78.1		78.6	76.2	71.4	0.82[c]
past	5.7		5.6	6.0	7.1	
never	16.2		15.8	17.9	21.4	
Hypertension, %	62.4		59.6	72.6	92.9	<0.01
Dyslipidemia, %	47.8		46.2	53.6	64.3	0.08
Diabetes mellitus, %	25.8		25.1	23.8	64.3	0.06
Hemoglobin, g/dL	14.4	(1.6)	14.4	14.2	13.2	<0.01
K6 scale[d]	8.7	(3.0)	8.7	8.8	9.4	0.43
Serum creatinine, micromol/L	79.6	(18.6)	73.4	99.0	152.0	<0.01
mg/dL[e]	0.9	(0.2)	0.83	1.12	1.72	
Proteinuria, %						<0.01
no	61.1		62.9	57.1	28.6	
trace (-/+)	33.7		33.0	36.9	35.7	
positive (+)	5.2		4.1	6.0	35.7	

CASI score	89.8	(5.9)	90.2	88.0	85.6	<0.01

BMI, body mass index; CASI, Cognitive Abilities Screening Instrument; eGFR, estimated glomerular filtration rate (Estimates were computed using serum creatinine concentration according to the CKD Epidemiology Collaboration (CKD-EPI) Equation modified for the Japanese); HDL, high-density lipoprotein; K6, 6-item Kessler Psychological Distress Scale; LDL, low-density lipoprotein; NGSP, National Glycohemoglobin Standardization Program.

CASI score ranges from 0 to 100. Score <74 raises possibility of dementia); Hypertension: defined as systolic/diastolic blood pressure ≥140/90 mm Hg or medication use; Diabetes mellitus: defined as fasting glucose ≥ 7.0mmol/L (126 mg/dL) or HbA1c [NGSP] ≥6.5% or medication use; Dyslipidemia: defined as LDL-cholesterol ≥3.6mmol/L (140 mg/dL) or HDL-cholesterol <1.0mmol/L (40 mg/dL) or medication use.

[a]P-values were computed using linear regression treating eGFR category as ordinal for continuous variable, and by Mantel Haenzel test for linear trend or Fisher's exact test if appropriate for categorical variable.

[b]Conversion unit for serum low-density cholesterol and high-density cholesterol from mmol/L to mg/dL is 38.61.

[c]P-value was obtained using Fisher's exact test.

[d]Four participants had missing scales.

[e]Conversion unit for serum creatinine from micromol/L to mg/dL is 0.0113.

Online Supplementary Material

eTable 2. Multivariable-adjusted mean slope of CASI score in men aged ≥65 years who were free of stroke (N=561, 2009–2014, Shiga, Japan)

	Adjusted mean difference in CASI score				*P*
	mean	95% CI			
Chronic kidney disease, present	-1.26	-2.14	,	-0.38	<0.01
Age, per 1 year	-0.38	-0.49	,	-0.28	<0.01
Education, per 1 year	0.76	0.58	,	0.95	<0.01
Smoking (ref. never)			,		
current	-0.46	-1.96	,	1.03	0.54
former	0.21	-0.92	,	1.34	0.71
Drinking (ref. never)			,		
current	0.78	-0.43	,	1.99	0.21
former	-0.60	-2.74	,	1.54	0.58
Body mass index, per 1 kg/m^2	-0.01	-0.17	,	0.16	0.92
Hypertension, present	-0.64	0.31	,	-1.58	0.19
Dyslipidemia, present	0.74	1.66	,	-0.17	0.11
Diabetes, present	-0.40	0.63	,	-1.42	0.45
Hemoglobin, per 1 g/dL	0.05	-0.24	,	0.34	0.73

CASI, the Cognitive Abilities Screening Instrument; CI, confidence interval; CKD, chronic kidney disease.

CKD was defined as either estimated glomerular filtration rate (eGFR) < 60 mL/min/1.73m^2 or presence of trace or greater degree of proteinuria. Hypertension was defined as systolic/diastolic blood pressure ≥140/90 mm Hg or medication use; Diabetes mellitus was defined as fasting glucose ≥ 7.0mmol/L (126 mg/dL) or HbA1c [NGSP] ≥6.5% or medication use; Dyslipidemia was defined as LDL-cholesterol ≥3.6mmol/L (140 mg/dL) or HDL-cholesterol <1.0mmol/L (40 mg/dL) or medication use. eGFR was based on serum creatinine concentration according to the CKD Epidemiology Collaboration (CKD-EPI) Equation modified for the Japanese.

Online Supplementary Material

eTable 3. Crude and adjusted mean CASI score according to four categories by proteinuria and eGFR in men aged ≥65 years who were free of stroke (N=561, 2009–2014, Shiga, Japan)

	Crude CASI score	Adjusted CASI score (95% confidence interval)				P-value for pairwise comparison		
						isolated proteinuria	isolated low eGFR	proteinuria and low eGFR
No CKD (n=291)	90.4	90.4	(89.8	,	91.0)	0.10	0.10	<0.01
Isolated proteinuria (n=172)	89.9	89.5	(88.7	,	90.3)	-	0.60	0.02
Isolated low eGFR (n=52)	88.7	89.1	(87.6	,	90.5)	-	-	0.14
Proteinuria and low eGFR (n=46)	86.5	87.5	(85.9	,	89.1)	-	-	-

CASI, the Cognitive Abilities Screening Instrument; eGFR, estimated glomerular filtration rate (Estimates were computed using serum creatinine concentration according to the CKD Epidemiology Collaboration (CKD-EPI) Equation modified for the Japanese).

Proteinuria defined as having trace (-/+) or more of proteinuria by dipstick, low eGFR as eGFR <60 mL/min/1.73m^2. Each of the four categories, “No CKD”, “Isolated proteinuria”, “Isolated low eGFR”, and “Proteinuria and low eGFR”, was treated as nominal in linear regression model. The set of adjusting covariates was same as in the main analyses: age (years), highest education attained (years), drinking/smoking habit (current/past/never), body mass index (kg/m^2), hypertension (yes/no), diabetes mellitus (yes/no), dyslipidemia (yes/no), and hemoglobin (g/dL).

Online Supplementary Material

eTable 4. Crude and multivariable-adjusted mean CASI score according to proteinuria and/or eGFR (by 2012 Japanese Society of Nephrology equation) category in men aged ≥65 years who were free of stroke (N=561, 2009–2014, Shiga, Japan)

		n	crude score mean	crude score (SD)	Single adjustment[a] score (95% CI)		trend *P*	Mutual adjustment[b] score (95% CI)		trend *P*
Proteinuria	(-)	343	90.1	(5.4)	90.2 (89.6 , 90.7)			90.2 (89.6 , 90.7)		
	(±)	189	89.3	(6.7)	89.2 (88.5 , 90.0)		0.011	89.3 (88.5 , 90.0)		0.020
	≥(1+)	29	88.2	(6.7)	88.1 (86.2 , 90.1)	*		88.3 (86.3 , 90.3)		
eGFR[c], ml/min/1.73m^2	≥60	404	90.2	(5.6)	90.1 (89.6 , 90.6)			90.1 (89.5 , 90.6)		
	59-40	142	88.8	(6.5)	89.0 (88.1 , 89.9)	*	0.022	89.0 (88.1 , 89.9)	*	0.042
	<40	15	86.0	(7.9)	88.3 (85.5 , 91.0)			88.8 (86.0 , 91.6)		

CASI, the Cognitive Abilities Screening Instrument (scored 0-100, with score <74 raising possibility of dementia); CI, confidence interval; eGFR, estimated glomerular filtration rate; SD, standard deviation.

In all adjustment, the following covariates were included: age (years), highest education attained (years), drinking/smoking habit (current/past/never), body mass index (kg/m^2), hypertension (yes/no), diabetes mellitus (yes/no), dyslipidemia (yes/no) and hemoglobin (g/dL). Hypertension: defined as systolic/diastolic blood pressure ≥140/90 mmHg or medication use; Diabetes mellitus: defined as fasting glucose ≥ 7.0mmol/L (126mg/dL) or HbA1c [NGSP] ≥6.5% or medication use; Dyslipidemia: defined as LDL-cholesterol ≥3.6mmol/L (140mg/dL) or HDL-cholesterol <1.0mmol/L (40mg/dL) or medication use.

[a]In single adjustment model, either proteinuria (no/trace/≥1+) or eGFR-category (<40/40-59/≥60 mL/min/1.73m^2) was included.

[b]In mutual adjustment model, both proteinuria and eGFR were included. [c]Estimates were computed using serum creatinine concentration according to the formula proposed by the Japanese Society of Nephrology 2018.

*Statistically significant (P<0.05) as compared to normal category (either no proteinuria or eGFR ≥60mL/min/1.73 m^2).

Online Supplementary Material

eTable 5. Crude and multivariable-adjusted mean CASI score according to proteinuria and/or eGFR category (using cutoff of 45) in men aged ≥65 years who were free of stroke (N=561, 2009–2014, Shiga, Japan)

		n	crude score mean	crude score (SD)	Single adjustment[a] score (95% CI)		trend *P*	Mutual adjustment[b] score (95% CI)		trend *P*
Proteinuria	no	343	90.1	(5.4)	90.2 (89.6 , 90.7)			90.2 (89.6 , 90.7)		
	trace	189	89.3	(6.7)	89.2 (88.5 , 90.0)		0.011	89.3 (88.5 , 90.0)		0.021
	≥(1+)	29	88.2	(6.7)	88.1 (86.2 , 90.1)	*		88.1 (86.2 , 90.1)		
eGFR[c], mL/min/1.73 m²	≥60	463	90.2	(5.5)	90.1 (89.6 , 90.5)			90.0 (89.5 , 90.5)		
	59-45	74	87.5	(7.4)	87.9 (86.7 , 89.1)	*	0.032	88.0 (86.8 , 89.2)	*	0.069
	<45	24	87.9	(7.0)	89.6 (87.5 , 91.8)			90.1 (87.9 , 92.2)		

CASI, the Cognitive Abilities Screening Instrument; CI, confidence interval; eGFR, estimated glomerular filtration rate; SD, standard deviation.

In all adjustment, the following covariates were included: age (years), highest education attained (years), drinking/smoking habit (current/past/never), body mass index (kg/m²), hypertension (yes/no), diabetes mellitus (yes/no), dyslipidemia (yes/no) and hemoglobin (g/dL). Highlighted area in light blue indicates the values that should not change regardless of eGFR cutoff of either 45 or 30 mL/min/1.73 m² (see **Table 2** in the main text).

[a]In single adjustment model, either proteinuria (no/trace/≥1+) or eGFR-category (<45/45-59/≥60 mL/min/1.73m²) was included.

[b]In mutual adjustment model, both proteinuria and eGFR were included.

[c]Estimates were computed using serum creatinine concentration according to the CKD Epidemiology Collaboration (CKD-EPI) Equation modified for the Japanese.

*Statistically significant (P<0.05) as compared to normal category (either no proteinuria or eGFR ≥60mL/min/1.73 m²)

Online Supplementary Material

eAppendix 1. Members of the Shiga Epidemiological Study of Subclinical Atherosclerosis (SESSA) Research Group

Co-chairpersons: Hirotsugu Ueshima,and Katsuyuki Miura (Department of Public Health, Center for Epidemiologic Research in Asia, Shiga University of Medical Science, Otsu, Shiga).

Research members: Minoru Horie, Yasutaka Nakano, Takashi Yamamoto (Department of Cardiovascular and Respiratory Medicine, Shiga University of Medical Science, Otsu, Shiga), Emiko Ogawa (Health Administration Center, Shiga University of Medical Science, Otsu, Shiga), Hiroshi Maegawa, Itsuko Miyazawa (Division of Endocrinology and Metabolism, Department of Medicine, Shiga University of Medical Science, Otsu, Shiga), Kiyoshi Murata (Department of Radiology, Shiga University of Medical Science, Otsu, Shiga), Kenichi Mitsunami (Shiga University of Medical Science, Otsu, Shiga), Kazuhiko Nozaki (Department of Neurosurgery, Shiga University of Medical Science, Otsu, Shiga), Akihiko Shiino (Molecular Neuroscience Research Center, Shiga University of Medical Science, Otsu, Shiga), Isao Araki (Kusatsu Public Health Center, Kusatsu, Shiga), Teruhiko Tsuru (Department of Urology, Shiga University of Medical Science, Otsu, Shiga), Ikuo Toyama (Unit for Neuropathology and Diagnostics, Molecular Neuroscience Research Center, Shiga University of Medical Science, Otsu, Shiga), Hisakazu Ogita, Souichi Kurita (Division of Medical Biochemistry, Department of Biochemistry and Molecular Biology, Shiga University of Medical Science, Otsu, Shiga), Toshinaga Maeda (Central Research Laboratory, Shiga University of Medical Science, Otsu, Shiga), Naomi Miyamatsu (Department of Clinical Nursing Science Lecture, Shiga University of Medical Science, Otsu, Shiga), Toru Kita (Kobe Home Care Institute, Kobe, Hyogo), Takeshi Kimura (Department of Cardiovascular Medicine, Kyoto University, Kyoto), Yoshihiko Nishio (Department of Diabetes, Metabolism, and Endocrinology, Kagoshima University, Kagoshima), Yasuyuki Nakamura (Department of Food Science and Human Nutrition, Faculty of Agriculture, Ryukoku University, Otsu, Shiga), Tomonori Okamura (Department of Preventive Medicine and Public Health, School of Medicine, Keio University, Tokyo), Akira Sekikawa, Emma JM Barinas-Mitchell (Department of Epidemiology, Graduate School of Public Health, University of Pittsburgh, Pittsburgh, PA, USA), Daniel Edmundowicz (Department of Medicine, Section of Cardiology, School of Medicine, Temple University, Philadelphia, PA, USA), Takayoshi Ohkubo (Department of Hygiene and Public Health, Teikyo University School of Medicine, Tokyo), Atsushi Hozawa (Preventive Medicine, Epidemiology Section, Tohoku University, Tohoku Medical Megabank Organization, Sendai, Miyagi), Nagako Okuda (Department of Health and Nutrition,

University of Human Arts and Sciences, Saitama), Aya Higashiyama (Research and Development Initiative Center, National Cerebral and Cardiovascular Center, Suita, Osaka), Shinya Nagasawa (Department of Epidemiology and Public Health, Kanazawa Medical University, Kanazawa, Ishikawa), Yoshikuni Kita (Faculty of Nursing Science, Tsuruga Nursing University, Tsuruga, Fukui), Yoshitaka Murakami (Division of Medical Statistics, Department of Social Medicine, Toho University, Tokyo), Aya Kadota (Center for Epidemiologic Research in Asia, Department of Public Health, Shiga University of Medical Science, Otsu, Shiga), Akira Fujiyoshi, Naoyuki Takashima, Takashi Kadowaki, Sayaka Kadowaki (Department of Public Health, Shiga University of Medical Science, Otsu, Shiga), Robert D. Abbott, Seiko Ohno, Maryam Zaid (Center for Epidemiologic Research in Asia, Shiga University of Medical Science, Otsu, Shiga), Hisatomi Arima (Department of Preventive Medicine and Public Health, Faculty of Medicine, Fukuoka University), Takashi Hisamatsu (Department of Environmental Medicine and Public Health, Faculty of Medicine, Shimane University), Naoko Miyagawa, Sayuki Torii, Yoshino Saito, Sentaro Suzuki and Takahiro Ito (Department of Public Health, Shiga University of Medical Science, Otsu, Shiga).
